# The effect of the rotator interval on glenohumeral kinematics during abduction

**DOI:** 10.1186/s12891-016-0898-x

**Published:** 2016-01-28

**Authors:** Babak Haghpanah, Kempland C. Walley, Andreas Hingsammer, Ethan R. Harlow, Ramin Oftadeh, Ashkan Vaziri, Arun J. Ramappa, Joseph P. DeAngelis, Ara Nazarian

**Affiliations:** Center for Advanced Orthopaedic Studies, Beth Israel Deaconess Medical Center, Harvard Medical School, 330 Brookline Avenue, RN115, Boston, MA 02215 USA; Carl J. Shapiro Department of Orthopaedic Surgery, Beth Israel Deaconess Medical Center, Harvard Medical School, Boston, MA USA; Department of Mechanical and Industrial Engineering, Northeastern University, Boston, MA USA

**Keywords:** Glenohumeral joint, Rotator interval translation, Kinematics, Shoulder laxity

## Abstract

**Background:**

The rotator interval (RI) has been exploited as a potentially benign point of entry into the glenohumeral (GH) joint. Bounded by the supraspinatus, subscapularis and coracoid process of the scapula, the RI is believed to be important in the shoulder’s soft tissue balancing and function. However, the role of the RI in shoulder kinematics is not fully understood. The purpose of this study is to describe the effect of the RI on GH motion during abduction of the arm.

**Methods:**

Six shoulders from three cadaveric torsos were studied to assess the impact of changes in the RI during abduction under four conditions: Intact (Baseline), Opened, Repaired (repaired with side-to-side tissue approximation, no overlap) and Tightened (repaired with 1 cm overlap). For each group, the GH translation and area under the Curve (AUC) were measured during abduction using an intact cadaveric shoulder (intact torso).

**Results:**

GH kinematics varied in response to each intervention and throughout the entire abduction arc. Opening the RI caused a significant change in GH translation. The Repair and Tightened groups behaved similarly along all axes of GH motion.

**Conclusions:**

The RI is central to normal GH kinematics. Any insult to the tissue’s integrity alters the shoulder’s motion throughout abduction. In this model, closing the RI side-to-side has the same effect as tightening the RI. Since suture closure may offer the same benefit as tightening the RI, clinicians should consider this effect when treating patients with shoulder laxity. This investigation provides an improved perspective on the role of the RI on GH kinematics during abduction. When managing shoulder pathology, surgeons should consider how these different methods of RI closure affect the joint’s motion. In different circumstances, the surgical approach to the RI can be tailored to address each patient’s specific needs.

## Background

The rotator interval (RI) is a triangularly shaped space bounded by the supraspinatus superiorly, the subscapularis inferiorly and the coracoid process of the scapula medially. It represents a complex interaction of the coracohumeral ligament, the superior glenohumeral (GH) ligament, GH joint capsule, and the supraspinatus and subscapularis tendons. The lateral apex of this triangle is composed of the intertubercular sulcus and transverse ligament and the coracoid process composes its base. The RI also contains the intra-articular portion of the long head of the biceps tendon and is concealed by a fibrous capsule [1]. The RI functionally limits external rotation and resists humeral head translation in the adducted shoulder and posterior translation in the flexed or abducted *and* externally rotated shoulder [2, 3]. In patients with shoulder instability this triangular area of connective tissue has been implicated in the treatment of various shoulder condition. Since the rotator cuff tendons are not injured, the RI provides an easy site for accessing the GH joint for many common shoulder procedures and is an ideal approach for the open treatment of septic arthritis. Recent interest in less invasive techniques has illustrated how this potential space can be exploited for shoulder arthroplasty [4]. In this way, the RI is believed to provide a safe point of entry to the shoulder with minimal impact on the joint’s kinematics.

However, in patients with shoulder instability, tightening the RI has been shown to decrease external rotation and eliminate the sulcus sign associated with joint laxity. This finding suggests that the RI has a more complex role in normal motion.

Few studies have examined the effect of the RI on shoulder kinematics. Of note, studies have investigated GH and scapulothoracic motion in normal patients and resultant kinematics in patients with shoulder instability or rotator cuff (RC) tears; however, the kinematics associated with RI insult and subsequent repair is uncertain.

The purpose of this study was to examine the impact of RI on GH kinematics during abduction using a cadaveric model with an intact GH and scapulothoracic articulation. Specifically, the shoulder kinematics with the RI intact (baseline) was compared to three experimental conditions: RI opened, RI repaired using a simple side-to-side approximation, and RI tightened by overlapping the tissue one centimeter. *We hypothesized that opening the RI would release the shoulder’s natural soft-tissue tension, resulting in increased GH translation during abduction of the arm; while closing the RI would return the motion to normal; and that overtightening of the RI would decrease translation of the joint during abduction, limiting GH translation relative to the baseline.*

## Methods

### Testing apparatus

**Fig. 1:**
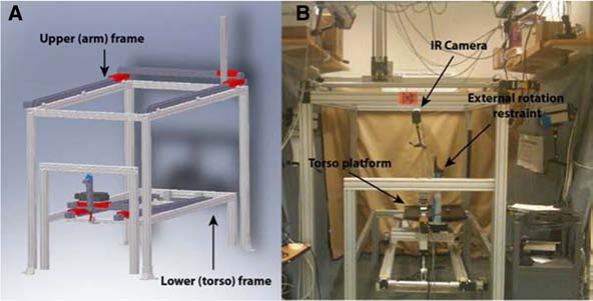
Robotic testing system that generates automated motion segments for a cadaveric torso over a designated trajectory. The seven degrees of freedom testing apparatus was designed and manufactured with four actuators on the lower frame to move the torso and with three actuators on the upper frame to move the hand with an additional rotational axis added to the lower frame to rotate the torso. **a** Apparatus schematic. **b** Apparatus photograph

A validated robotic system was used to generate automated motion trajectories for a cadaveric torso [5, 6]. The system consists of a lower frame holding the cadaveric torso and an upper frame to which the upper limb is attached (Fig. 1a and b). The lower (torso) frame can generate three translational degrees of freedom (DOF) along the x, y, and z-axes and one rotational DOF around the z-axis, while the upper (limb) frame has three translational DOFs along the x, y, and z-axes. Any desired motion trajectory within the range of the system is generated by linear and rotary closed loop actuators and controlled via a programmable central controller with high reproducibility and accuracy [5, 6].

### Cadaveric torsos and surgical procedures

Six shoulders from three fresh-frozen human cadavers were acquired (Medcure, Inc., Portland, OR, USA). The cadaveric torsos originated from three Caucasian males with an age of 55 ± 4 years, height of 190 ± 4 cm, and body mass index of 27.1 ± 1.85 kg/m^2^. The range of motion and laxity of the each shoulder was tested prior to testing procedures to see if the motion was normal without any reduction during the arc of abduction. Specimens were allowed to thaw at room temperature and tested immediately thereafter. The experimental protocol was performed sequentially, allowing each specimen to serve as its own control. Four conditions were compared:

1. Intact-A baseline for the data was established using the intact specimen.
2. Opened-The RI was cut, separating the leading edge of the supraspinatus from the superior edge of the subscapularis. The incision extended from the base of the coracoid to the humeral head (approximately 2–3 cm) in line with fibers of the rotator cuff.
3. Repaired-The RI was repaired with a running suture (Ethibond #1, Ethicon, Somerville, NJ, USA) to create no overlap or tightening. The arm was abducted, in a resting position, in twenty degrees of external rotation to prevent overtightening.
4. Tightened-the running suture was removed. The arm was allowed to rest in the neutral position. A new suture was then used to tighten the rotator interval using a horizontal mattress technique (Ethibond #1, Ethicon, Somerville, NJ, USA). The two passes were made with the needle separated by one centimeter. The needle entered one edge of the RI one centimeter from the cut edge and exited the other side one centimeter from the cut edge. The second pass was made in the opposite direction and the two suture limbs were tied, “tightening” the RI by two centimeters.
5. The running suture was removed and the RI was re-approximated with a horizontal mattress suture. The suture needle was passed one centimeter from the free edge of the tissue edge to tighten the RI by 2 cm.

### Simulation of abduction motion

Torsos were mounted on a rod fixture (Fig. 1a and b) and held in place with expanding foam [6, 7]. The hand was disarticulated at the distal radioulnar joint, and the arm was secured directly to the upper frame using a Schanz. The skin and the deltoid muscle were removed. Passive retro-reflective marker clusters were placed in the humeral shaft, the posterolateral acromion, and the sternum [6, 7]. To protect the specimens, testing was performed at a reduced speed (duration of motion, 28.6 s), in accordance with previous studies [6, 8]. The arm was raised in the coronal plane from 30 to 150° of abduction for three repetitions.

### Motion analysis

**Fig. 2:**
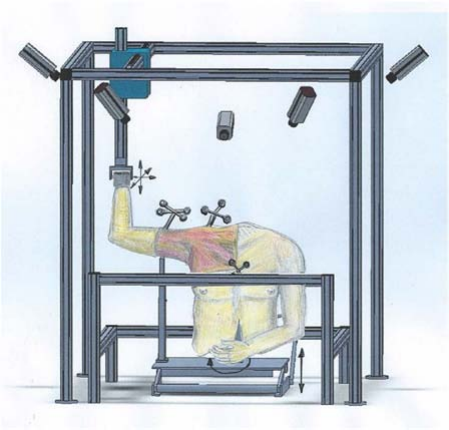
An illustration of a cadaveric torso mounted onto the lower frame of the testing system and the arm attached to the upper frame

**Fig. 3:**
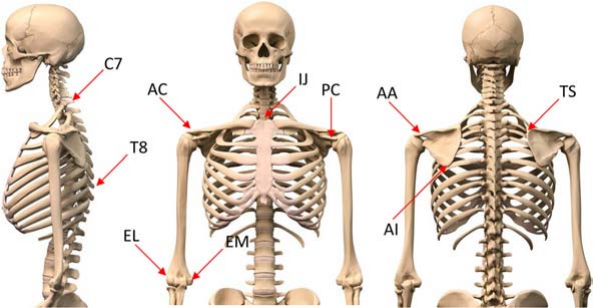
An illustration of the anatomic scapular, humeral, and thoracic landmarks were calibrated with respect to these technical (bone-embedded) markers, as defined by the International Society of Biomechanics: AA: angulus acromialis, AC: acromioclavicularis, AI: angulus inferior, C7: processus spinosus cervical 7, EL: epicondylus lateralis, EM: epicondylus medialis, IJ: incisura jugularis, PC: precessus corracoideus, TS: trigonum spinae and T8: processus spinosus thoracal 8

Five Qualisys Pro Reflex (Qualisys AB, Göteborg, Sweden) high-speed cameras (120 Hz) were used to record the motion of the passive retro-reflective bone-embedded marker clusters. The clusters were placed into the humeral shaft, the sternum, and the acromion (Fig. 2). Before testing, the cameras were subject to multi-aspect calibration [9]. Anatomic scapular, humeral, and thoracic landmarks were calibrated with respect to these technical (bone-embedded) markers, as defined by the International Society of Biomechanics (AC joint (AC), the posterolateral edge of the acromion (AA), the coracoid process (PC), the inferior angle of the scapula (AI), the root of the spine of the scapula (TS), the spinous process of the seventh cervical vertebra (C7) and eighth thoracic vertebra (T8), the xiphoid process, the suprasternal notch (IJ), and the medial and lateral epicondyles (EM and EL)) (Fig. 3) [9]. The calibrated scapular and humeral landmarks were analyzed per Meskers et al. to determine the instant center of rotation of the GH joint within the scapular reference system [10]. For all displacements, the x-axis, y-axis, and z-axis correspond to anterior-posterior (AP; coronal), superior-inferior (SI; sagittal), and medial-lateral (M; transverse) planes, respectively. Displacements were quantified in mm.

In this system, the z-axis is a line connecting the TS and AA points; the x-axis originates from the AA point and is perpendicular to the plane formed by the AI, the AA, and the TS points; and the y-axis is the common line perpendicular to the x- and z-axes.

### Statistical analysis

GH translation was recorded continuously throughout the abduction motion from 30 to 150°. For each condition, the average of three repetitions of GH translation was plotted over time to calculate the total translation and the area under the curve (AUC) for each motion segment. Absolute GH translation was calculated for Baseline, Opened, Repaired and Tightened conditions. A mixed model analysis of variance (ANOVA) was used to compare GH translations for each condition on each axis. AUC was calculated using the trapezoidal rule to appropriately assess the path-dependent motion (MATLAB version 12, MathWorks, Natick, MA, USA). The Wilcoxon signed–rank test was used to compare the AUC.

Six specimens allowed for the detection of a difference of greater than 1.0 mm in GH translation and 85 % power to detect mean differences of greater than 1.2 mm translation using ANOVA with a compound symmetry correlation structure to handle the paired specimens.

Cadaveric studies do not require Institutional review Board consideration or approval at our institution. Statistical analysis was conducted using SPSS (version 21.0, IBM-SPSS, Armonk, NY, USA). Two-tailed *p*-values less than 0.05 were considered significant.

## Results

**Fig. 4:**
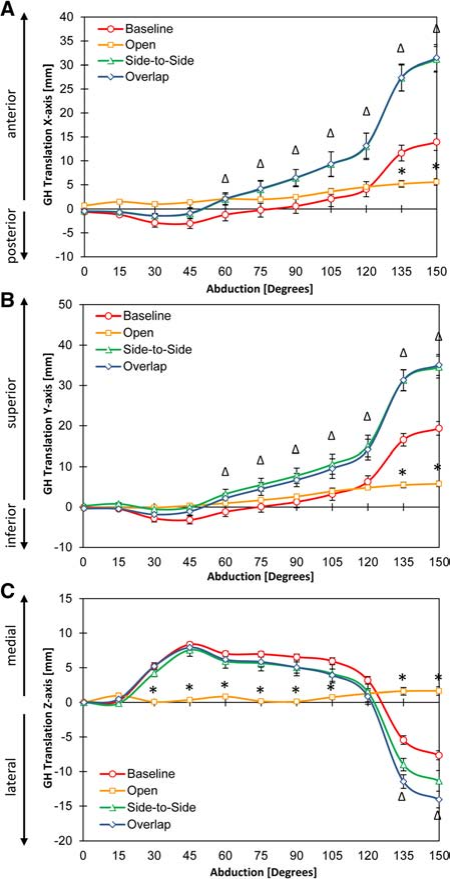
**a**-**c** Glenohumeral (GH) translations in the coronal plane (x-axis), sagittal plane (y-axis), and transverse (z-axis) planes, respectively. Four study groups: baseline, rotator interval 2 cm insult (OPEN), side-by-side repair (Side-to-Side) and tightened rotator interval repair (Overlap) are identified by dark blue, cyan, yellow and red lines, respectively. Symbol (*) denotes data significance (*p* ≤ 0.005) following insult (Open) of the rotator interval compared to Baseline. Symbol (◊) denotes data significance (*p* ≤ 0.005) following both mechanisms of repair (Side-to-Side, Overlap) compared to Baseline

Differences were observed in GH translations in the coronal plane (x-axis) between Baseline and Opened conditions at 135° and 150° (both *p* < 0.05; Fig. 4a, *), and between both closed conditions (Repaired, and Tightened) and Baseline at 60°, 75°, 90°, 105°, 120°, 135°, and 150° (all *p* < 0.05; Fig. 4a, ^∆^).

In the sagittal plane (y-axis), Opened and Baseline were significantly different at 135° and 150° (both *p* < 0.05; Fig. 4b, *). Moreover, differences in GH translation in the sagittal plane were observed between both closed conditions (Repaired and Tightened) and Baseline at 60°, 75°, 90°, 105°, 120°, 135°, and 150° (all *p* < 0.05; Fig. 4b, ^∆^).

In the transverse plane (z-axis), GH translation was different between Opened and Baseline at 30°, 45°, 60°, 75°, 90°, 105°, 120°, 135°, and 150° (all *p* < 0.05; Fig. 4c, *). Finally, both closed conditions (Repaired and Tightened) demonstrated significant differences at 135° and 150° when compared to Baseline (both *p* < 0.05; Fig. 4c, ^∆^).

**Fig. 5:**
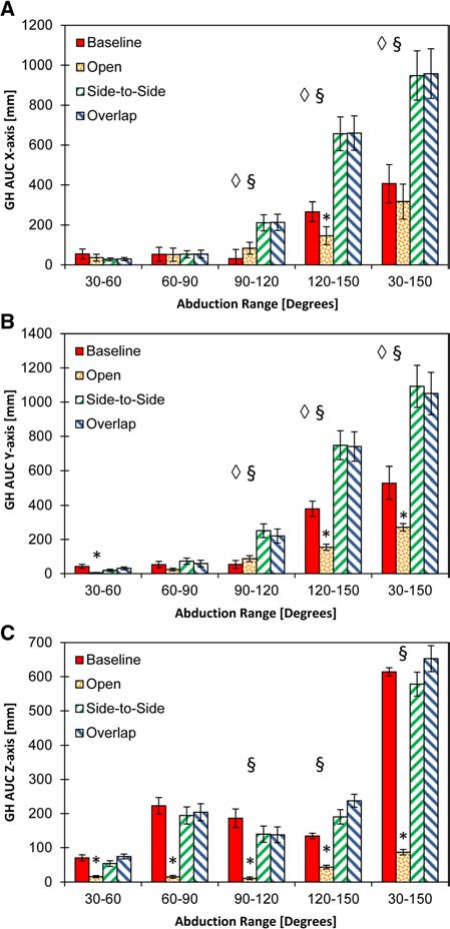
**a**-**c** Glenohumeral (GH) AUC in the coronal plane (x-axis), sagittal plane (y-axis), and transverse (z-axis) planes, respectively. Four study groups: baseline, rotator interval 2 cm insult (OPEN), side-by-side repair (Side-to-Side) and tightened rotator interval repair (Overlap) are identified by dark blue, cyan, yellow and red lines, respectively. Symbol (*) denotes data significance (*p* ≤ 0.005) following insult (Open) of the rotator interval compared to Baseline. Symbol (◊) denotes data significance (*p* ≤ 0.005) following both mechanisms of repair (Side-to-Side, Overlap) compared to Baseline. Symbol (§) denotes data significance (*p* ≤ 0.005) between side-by-side repair (Side-to-Side) and tightened rotator interval repair (Overlap) and insult (Open) of the rotator interval when compared to Baseline

GH AUC in the transverse plane demonstrated differences between Open and Baseline at 120°-150° (*p* < 0.05; Fig. 5a *). GH AUC in the transverse plane demonstrated differences between both Repaired and Tightened when compared to Baseline (⋄) and Opened (§) at 90°-120°, 120°-150°, and for the entire abduction 30°-150° range (all *p* < 0.05; Fig. 5a). GH AUC in the sagittal plane revealed differences between Opened and Baseline states at 30°-60°, 120°-150°, and for the entire abduction 30°-150 (*p* < 0.05; Fig. 5b, *). GH AUC was significantly different in the sagittal plane for Repaired and Tightened when compared to Baseline (⋄) and Opened (§) at 90°-120°, 120°-150°, and for the entire abduction 30°-150° (all *p* < 0.05; Fig. 5b). GH AUC in the transverse plane was different for Opened versus Baseline at 30°-60°, 60°-90°, 90°-120°, 120°-150°, and for the entire abduction 30°-150° (all *p* < 0.05; Fig. 5c). AUC in the transverse plane was also different for Repaired and Tightened when compared to Opened (§) at 90°-120°, 120°-150°, and for the entire abduction 30°-150° (all p > 0.05; Fig. 5b). AUC analysis did not show a difference between Repaired and Tightened at 90°-120°, 120°-150° (both p > 0.05; Fig. 5c).

## Discussion

The RI resists inferior and posterior translation in the abducted shoulder, particularly when the arm is externally rotated [3, 11–17]. This constraint results from the RI’s stabilizing effect on GH motion, preventing displacement of the humeral head while connecting the supraspinatus to the subscapularis [3]. Generalized tissue laxity can negate this effect, resulting in multidirectional instability (MDI). For MDI, arthroscopic RI closure, combined with capsular plication, has been used to increase shoulder stability while reducing the range of motion [18–20].

However, the role of the RI in GH kinematics is still debated. Provencher et al*.* found that arthroscopic RI closure added little support to posterior and inferior stability of the shoulder during abduction [21]. Harryman et al*.* described how a medial-to-lateral open RI imbrication decreased inferior translation in an abducted shoulder and decreased posterior translation in a flexed shoulder [3]. Decreased anterior GH translation occurs consistently following RI closure [21–23].

At the same time, RI closure can overtighten the shoulder, constraining motion at some arm positions [20]. Though inconsistent, the data gathered from cadaveric RI closure suggest that this tissue has an effect on shoulder kinematics.

Few studies have delineated the effect of opening the RI on GH kinematics. This study sought to characterize how opening the RI may uncouple the stabilizing effect of the anterior and superior tissues, and whether it’s subsequent closure would restore the shoulder’s kinematics in this cadaveric model.

Four conditions were compared and the GH displacements during continuous abduction were recorded using a validated, automated upper extremity testing system with seven degrees of freedom. Glenohumeral kinematics was disturbed throughout abduction following RI manipulation. Both side-to-side closure and tightening altered the shoulder’s kinematics equivalently throughout all angles of abduction and resembled the kinematics of the baseline profile from 30–90° of abduction. This effect suggests that the RI plays an important role in regulating the shoulder’s motion as a mechanical support.

The equivalence of the two repair techniques on GH motion from 30 to 90° of abduction is clinically insightful. In treating shoulder instability, tightening the RI may be the ideal approach because it does not negatively affect the shoulder’s motion as this experiment demonstrates. Additionally, it produces a more widespread stabilizing effect on the shoulder joint than the side-to-side repair.

With the exception of medial-lateral displacement, the AUC of GH translation varied significantly from 90–150° of abduction when the baseline and repair conditions were compared. These data suggest that RI’s contribution to shoulder kinematics may be altered when the arm is abducted more than 90°. Large changes in shoulder kinematics were observed after both methods of RI closure, and questions the use of RI closure as the sole method of treatment for shoulder instability.

This study is not without limitations. First, we examined passive abduction in a cadaveric model. Dynamic forces central to glenohumeral stability and shoulder motion, including various degrees of rotation relating to physiologic GH motion, were not simulated [24]. Secondly, the size of the RI opening size was not studied. It was widely incised to separate the supraspinatus and infraspinatus from the base of the coracoid to the insertion on the humerus. Varying the length of the RI opening could influence the extent of change from baseline. Simply, a smaller incision may produce less change. Thirdly, GH translation was calculated using a regression analysis to determine the instant center of rotation using defined anatomical landmarks. The precision of this characterization depends on the quality of the anatomic calibration [6, 8] As a result of this estimation, there is inherent variability, as anatomical landmarks are areas rather than discrete points [25]. This effect may partly explain the inter-variability among specimens. The speed of the abduction from motion from 30° to 120° was simulated at a reduced speed than what may be considered clinically relevant. While Bergmann et al. [26] have demonstrated that reduced speeds change GH peak forces and corresponding moments, the directions of GH forces remain constant. Furthermore, glenohumeral forces were not calculated to further distinguish between the methods of RI closure. Due to the fact that this is a cadaveric study, it is impossible to predict the exact strength and functional outcome of repaired tissues. This simulated parametric biomechanical study can most assertively investigate the strength and functional outcome of suture materials and suture technique in the groups tested and serves as a physiologic approximation of shoulder kinematics. Moreover, the physiological organization of collagen and other matrix elements will change the characteristics of the tissues during the healing process whereas our results communicate affect immediately following RI insult and repair.

## Conclusions

Changes in the rotator cuff interval have a significant effect on glenohumeral kinematics during abduction. This investigation provides an improved perspective on the role of the RI on GH kinematics during abduction. This study demonstrates that GH translation is decreased with RI closure solely during abduction without rotation in a cadaveric torso specimen. The impact on clinical situations needs addition study measuring translation with abduction and rotation of the humerus. When managing shoulder pathology, surgeons should consider how these different methods of RI closure affect the joint’s motion. Suture closure may offer the same benefit as tightening the rotator interval. These findings may be relevant with regard to the treatment of shoulder laxity.
